# Links between facets of alexithymia, self-rated interoception, and social perception

**DOI:** 10.3389/fpsyg.2025.1669056

**Published:** 2026-01-09

**Authors:** Lorna S. Jakobson, Pauline M. Pearson, Sarah N. Rigby

**Affiliations:** 1 Department of Psychology, University of Manitoba, Winnipeg, MB, Canada; 2 Department of Psychology, University of Winnipeg, Winnipeg, MB, Canada

**Keywords:** alexithymia, embodiment, externally oriented thinking, facial expression, interoception, sensory processing sensitivity, social perception

## Abstract

In three studies we examined relationships between facets of alexithymia, sensory processing sensitivity (SPS), interoception, and social perception. In Study 1 (*N* = 145), sensitivity to subtle cues (a positive SPS trait) negatively mediated the link between having an external focus (externally oriented thinking) and reporting low subjective interoceptive accuracy. In Study 2 (*N* = 98), sensitivity to subtle cues negatively mediated links between having an external focus and the capacity to notice and sustain/control attention to body sensations and to connect these sensations to emotional states. Conversely, difficulty identifying feelings was linked to the tendencies to try to distract oneself from and to worry about uncomfortable sensations, with the latter link being mediated by sensitivity to unpleasant stimulation (a negative SPS trait). In Study 3 (*N* = 53), having an external focus predicted reporting a reduced ability to sustain and control attention to body sensations which, in turn, predicted lower ratings of the intensity of another’s nuanced facial expression. Having an external focus also predicted a weak tendency to listen to one’s body for insight, which predicted finding expressions less pleasant. Finally, the pleasantness of the expressions predicted how willing viewers were to approach the person depicted. By teasing apart the unique variance in aspects of interoception and social perception attributable to distinct facets of alexithymia and SPS, this work may have important implications for theory building and for the design and implementation of therapies intended to address difficulties associated with atypical interoceptive and/or social processing.

## 1 Introduction

Interoception is a multidimensional construct that refers to “the top-down and bottom-up processes by which an organism senses, interprets, and integrates signals from within itself and below the skin, across conscious and nonconscious levels” (Desmedt et al., 2023a, p. 12). Research exploring interoception and its relationship with variables such as emotion processing, mental and physical health, and social functioning has increased tremendously over the past two decades. In some of this work, objective measures of interoception, such as accuracy in tracking one’s heartbeat, have been collected. While studies using these objective measures have been foundational in capturing some important aspects of interoception, researchers may turn to subjective, self-report measures when they wish to capture other dimensions, including individuals’ beliefs and perceptions about their internal bodily states. These are important to study as they provide insights into interoceptive awareness and related psychological processes, as well as metacognitive aspects of interoception (e.g., confidence in one’s interoceptive accuracy) that may not be directly observable through objective tasks.

Self-report questionnaires capture specific dimensions of subjective interoception (Desmedt et al., 2022, 2023a). For example, the Interoceptive Accuracy Scale (IAS; Murphy et al., 2020) was designed to measure beliefs about one’s ability to monitor internal signals precisely and accurately. Another popular self-report measure of interoception is the Multidimensional Assessment of Interoceptive Awareness (MAIA; Mehling et al., 2012, 2018). Desmedt et al. (2022) noted that the items included in this measure fall into several distinct clusters. According to these authors, one cluster captures aspects of “adaptive interoception.” It includes items relating to one’s awareness of subtle bodily sensations (e.g., changes in breathing, muscle tension) and stimulation that makes one feel comfortable; the capacity to self-regulate by focusing on body sensations; and the tendencies to listen to your body for insight and to experience the body as safe and trustworthy. Items that tap into the extent to which one worries about or tries to distract oneself from unpleasant sensations, such as pain, form separate clusters (Desmedt et al., 2022) that may capture aspects of interoception that may be maladaptive (see below).

In the current series of studies, we explored factors that may contribute to individual differences in the dimensions of subjective interoception captured by the IAS and the MAIA-2. Our primary goal was to add to the literature linking a personality trait known as alexithymia to deficits in specific interoceptive processes assessed through self-report.

Alexithymia is characterized by difficulties identifying and describing feelings (DIF and DDF) that are often accompanied by externally oriented thinking (EOT) and an impoverished fantasy life (Jakobson et al., 2024; Nemiah, 1996). It has also been linked to atypicalities in interoception or body awareness (e.g., Brewer et al., 2016; Murphy et al., 2018; Trevisan et al., 2021). Pollatos and Herbert (2018) review several models that have been proposed to explain this link. The first set of models suggest that people with alexithymia have difficulty integrating their sense of self (the subject body) with how they are perceived by themselves and others (the object body), resulting in “pathological embodiment” (p. 326). The second set suggest that alexithymia may be characterized by disruption in aspects of embodied cognition, such as the ability to embody others’ emotions (“empathic embodiment”). The third set suggest that alexithymia is characterized by problems with multisensory integration that can disrupt predictive coding, leading to prediction errors that reduce body trust. It is argued that, if this disruption occurs, people with alexithymia may learn to rely more heavily on input from the external environment than on internal signals to understand their responses to emotionally salient stimuli, leading to the development of EOT. Importantly, according to this argument, EOT could be seen as a marker for a weak tendency to turn attention inward—something researchers refer to as a low *interoceptive propensity* (Murphy, 2022) or a weak *interoceptive attention bias* (Desmedt et al., 2023a). We adopt the former term in the current work. Characterizing EOT in this way bears some similarity to how Preece et al. (2017) conceptualize and measure this construct, although they emphasize a failure to attend to one’s subjective *feelings*, rather than to the specific exteroceptive and interoceptive cues that shape these experiences. It is important to note, however, that other researchers conceptualize EOT more broadly to include a tendency toward pragmatic thinking (Taylor et al., 2023).

Jakobson and Rigby (2021) described a link between EOT and low self-reported interoceptive accuracy (measured with the IAS). This association was mediated by two tendencies: failing to notice sensory stimulation and actively trying to seek it out. The authors argued that individuals who score high on EOT may routinely fail to direct attention inward and may also have difficulty exercising the top-down attentional control needed to actively maintain representations of weak sensations in working memory. These authors speculated that this could limit their conscious experience of how subtle sensory stimuli (which may not automatically capture attention) resonate in the body. If this is correct, one would predict that those scoring high in EOT would display an “ability deficit” (Preece et al., 2017, p. 344) that could interfere with important aspects of interoceptive processing including self-reported interoceptive accuracy and certain aspects of adaptive interoception (Desmedt et al., 2022).

In contrast to the above, past research has suggested an association between other facets of alexithymia (DIF and DDF) and discomfort arising from unpleasant internal signals or those associated with negative affective states such as anxiety (e.g., shortness of breath, thudding heartbeat, sweaty palms) (Betka et al., 2018; Longarzo et al., 2015). Hypersensitivity to bodily sensations that are perceived as uncomfortable or unpleasant, such as pain, could be maladaptive if it triggers and/or is reinforced by negative appraisals (e.g., “I’m afraid the pain will never go away”). This hypersensitivity may also lead those scoring high on DIF and DDF to worry about or try to ignore or distract themselves from unpleasant sensations, which in some circumstances could be maladaptive.

In the research described below, we sought to identify variables that might mediate relationships between specific facets of alexithymia and dimensions of subjective interoception measured by the IAS and the MAIA-2. We also explored links between alexithymia, subjective interoception, and performance on a behavioral task presumed to involve emotional contagion and embodied cognition—both of which are believed to depend on accurately processing and integrating interoceptive cues (Del Popolo Cristaldi et al., 2024).

## 2 Study 1

The key question we addressed in our first study was whether individual differences in the personality trait sensory processing sensitivity (SPS) might mediate the link between facets of alexithymia and self-reported interoceptive accuracy as measured by the IAS. Many people with alexithymia score high on SPS (Rigby et al., 2020; Jakobson and Rigby, 2021; Van Landeghem and Jakobson, 2024). People with SPS often report being highly sensitive to subtle bodily and environmental stimuli and to how aesthetic cues (e.g., a room’s lighting, music, the visual arts) resonate in their bodies at an emotional level (Attary and Ghazizadeh, 2021; De Gucht et al., 2022). These features belong to a “positive” SPS trait cluster (SPS+). However, highly sensitive people also frequently report being made to feel uncomfortable or overwhelmed by strong sensory stimulation and multitasking demands. These features belong to a “negative” SPS trait cluster (SPS–; Attary and Ghazizadeh, 2021; De Gucht et al., 2022). Whereas SPS+ traits are negatively related to EOT, SPS– traits are positively associated with DIF and DDF (Liss et al., 2008; Jakobson et al., 2024).

Drawing on the findings from Jakobson and Rigby (2021), we hypothesized that the strength of self-reported SPS+ traits would negatively mediate the relationship between EOT and interoceptive accuracy. Specifically, we expected to find that those reporting a weak interoceptive propensity (as indexed by high EOT scores) would report being less sensitive to subtle bodily sensations (low SPS+) and that this, in turn, would predict lower perceived ability to accurately monitor one’s internal cues (low IAS scores). Such a result would challenge the view that self-reported interoceptive propensity and accuracy are not associated (Gabriele et al., 2022; Murphy et al., 2020).

### 2.1 Materials and methods

#### 2.1.1 Participants

Data were collected from a convenience sample of 148 students enrolled in an Introduction to Psychology course, who earned credit toward a research participation option.^1^ After exclusions (see below) the final sample included 145 participants (75 females; *M*_age_ = 19.0 years, *SD* 2.8). This sample size was expected to provide adequate power to test our planned mediation model based on both the standard rule-of-thumb (as there were 49 cases per parameter) and the minimum sample size needed to detect full simple mediation with medium effect size suggested by Sim et al. (2022). A *post-hoc* analysis confirmed that the observed power for the mediation model tested was 0.75. The study protocol was approved by our institutional Research Ethics Board and all participants provided informed consent.

#### 2.1.2 Procedure and measures

Testing took place in a computer lab that could accommodate groups of up to 30 people at individual workstations. Participants provided demographic information (age, biological sex). They then completed (in counterbalanced order): (a) a set of self-report measures administered via the Qualtrics survey platform; and (b) the Adult/Adolescent Sensory Profile (Brown and Dunn, 2002), which was administered in paper format. Data on the latter measure were collected for a separate investigation. The measures administered in Qualtrics that were extracted for the current study are described below.

##### 2.1.2.1 Toronto Alexithymia Questionnaire

The Toronto Alexithymia Scale (TAS-20; Bagby et al., 1994) is a 20-item self-report questionnaire widely used in the assessment of alexithymia. It includes three subscales tapping into features that are characteristic of alexithymia including DIF (e.g., *I have physical sensations that even doctors do not understand*), DDF (e.g., *It is difficult for me to find the right words for my feelings*), and EOT (e.g., *I find examination of my feelings useful in solving personal problems* [reverse scored]). Participants rate the degree to which each item describes them on a 5-point Likert scale ranging from 1 (*strongly disagree*) to 5 (*strongly agree*). After reverse-scoring the five negatively keyed items, ratings are summed to yield three subscale scores and a total score, with higher scores reflecting stronger endorsement of alexithymic traits. The three-factor structure of this measure is reliable, well validated, and highly generalizable across languages and cultures (Parker et al., 2003a, 2003b). Furthermore, previous studies have shown the TAS-20 to have good internal consistency and test–retest reliability (Bagby et al., 1994).

##### 2.1.2.2 Measures assessing sensory processing sensitivity

Participants completed two complementary measures of SPS: the Highly Sensitive Person Scale (HSPS; Aron and Aron, 1997) and the short form of the Orienting Sensitivity subscale of the Adult Temperament Questionnaire (Evans and Rothbart, 2007). The 27 items that comprise the HSPS are rated on a 7-point Likert scale ranging from 1 (*not at all*) to 7 (*extremely*), with ratings averaged to produce a total score. Three subscales can be extracted (Smolewska et al., 2006). The Low Sensory Threshold subscale (6 items) taps into the extent to which one experiences unpleasant sensory arousal (e.g., *Are you particularly sensitive to the effects of caffeine*?). The Ease-of-Excitation subscale (12 items) measures the tendency to become easily overwhelmed by external and internal stimuli/demands (e.g., *Do you find it unpleasant to have a lot going on at once*?). The Aesthetic Sensitivity subscale (7 items) assesses one’s sensitivity to subtle, aesthetic features of the environment (e.g., *Do you notice and enjoy delicate or fine scents, tastes, sounds, work of art?*). Total scores on the HSPS are approximately normally distributed (Lionetti et al., 2018) and this measure has strong psychometrics (Aron and Aron, 1997; Smith et al., 2019).

The 15 items comprising the Orienting Sensitivity scale (OS) are responded to on a 7-point Likert scale ranging from 1 (*Extremely untrue of you*) to 7 (*Extremely true of you*). There are three (5-item) subscales. The Neutral Perceptual Sensitivity subscale assesses awareness of low-intensity stimuli from the body and the external environment (e.g., *I often notice mild odors and fragrances*). The Affective Perceptual Sensitivity subscale taps into awareness of how low-intensity environmental stimuli affect one’s mood (e.g., *I am often aware how the color and lighting of a room affects my mood*). The Associative Sensitivity subscale assesses the extent to which one engages in internal processes *not* driven by environmental stimuli, including imagery, dreaming, and aspects of problem solving (e.g., *When I am resting with my eyes closed, I sometimes see visual images*). The OS scale has good psychometric properties (Evans and Rothbart, 2007).

Previous work supports the view that the subscales of the HSPS and OS scales can be grouped into two clusters, with the Low Sensory Threshold and Ease-of-Excitation subscales loading onto a factor that reflects the strength of SPS– traits, and the Aesthetic Sensitivity and OS subscales loading onto a factor that reflects the strength of SPS+ traits (Jakobson et al., 2024). As such, we computed a composite score for each trait cluster by finding the mean score (out of 7) across relevant subscales. These composite scores were used in the present analyses.

##### 2.1.2.3 Interoceptive Accuracy Scale

The IAS (Murphy et al., 2020) is a unidimensional self-report measure of interoceptive accuracy. It includes 21 items that tap into one’s perception of a wide range of bodily sensations (e.g., *I can always accurately perceive when I am hungry*). Participants respond to each item using a five-point Likert scale ranging from 1 (*Strongly agree*) to 5 (*Disagree strongly*). Scores can range from 21 to 105, with higher scores representing better subjective interoceptive accuracy. The IAS exhibits good internal consistency, test–retest reliability, and construct validity (Murphy et al., 2020).

##### 2.1.2.4 Attention checks: Conscientious Responders Scale

The Conscientious Responders Scale (Marjanovic et al., 2014) is a measure designed to assist researchers in detecting poor effort in participants completing surveys. The five items are randomly dispersed throughout other survey items. Each item instructs participants to respond in a specific way (e.g., *To respond to this question, please choose option number five, “slightly agree.”*) There are five response options for each item, and responses are scored as correct or incorrect. Based on the authors’ recommendations, scores less than 3 are taken as evidence that the respondent is exhibiting poor effort and should be excluded.

### 2.2 Results

#### 2.2.1 Data cleaning and processing

A total of 148 individuals completed the study but three were excluded for failing to achieve an acceptable score on the Conscientious Responders Scale. There were no missing data in the final sample. All statistical analyses described below were run using SPSS software (v 29).

#### 2.2.2 Descriptive statistics and zero-order correlations between study variables

For the interested reader we provide descriptive statistics and zero-order correlations between study variables in Table 1. We focused on 12 correlations (shown in bold in the table) that were of particular interest. Reported below are the adjusted *p* values for these correlations, which were computed using the Benjamini-Hochberg correction to account for multiple comparisons.

**Table 1:** Study 1: descriptive statistics and zero-order correlations between study variables.

Measure	*M*	*SD*	TAS	DIF	DDF	EOT	SPS+	SPS–
TAS	50.4	12	--					
DIF	16.4	6.5	0.856***	--				
DDF	14.7	4.7	0.819***	0.610***	--			
EOT	19.4	4.4	0.578***	0.196*	0.258**	--		
SPS+	4.7	0.7	−0.089	**0.042**	0.036	−0.341***	--	
SPS–	3.9	0.9	0.286***	0.416***	0.216**	−0.068	0.212*	--
IAS	81.7	9.8	**−0.158**	−0.156	−0.104	−0.088	0.237**	**−0.084**

*N* = 145. Correlations of particular interest are in bold. Toronto Alexithymia Scale: TAS = total score; DIF = difficulty identifying feelings; DDF = difficulty describing feelings; EOT = externally oriented thinking. SPS+ and SPS– = positive and negative facets of sensory processing sensitivity, respectively. IAS = Interoceptive Accuracy Scale. *p < 0.05 without correction for multiple comparisons. **p < 0.01 without correction for multiple comparisons. ***p < 0.001 without correction for multiple comparisons.

There was a trend for high TAS-20 to be associated with low IAS scores (*p* = 0.086). As expected, TAS-20 subscale scores were correlated with one another (*p* ≤ 0.031) but DIF and DDF were more strongly associated with one another than with EOT. DIF and DDF were positively correlated with SPS– traits (*p* ≤ 0.018), but there was no significant relationship between SPS– traits and IAS scores. In contrast, as predicted, EOT was negatively correlated with SPS+ traits (*p* = 0.004), and scoring low on the SPS+ traits was associated with low subjective interoceptive accuracy (*p* < 0.001).

#### 2.2.3 Mediation analysis

EOT and IAS scores were not significantly correlated with one another. However, following recommendations by Hayes’ (2022) approach to mediation, we did not consider having a significant correlation between the predictor and the outcome variable a prerequisite for testing for mediation here or in our subsequent studies. Indeed, Hayes and Rockwood (2017) state that “…rejection of the null hypothesis that the indirect effect is zero (or an interval estimate that does not include zero) is sufficient to support a claim of mediation of the effect of X on Y through M” (p. 43).

**Figure 1:**
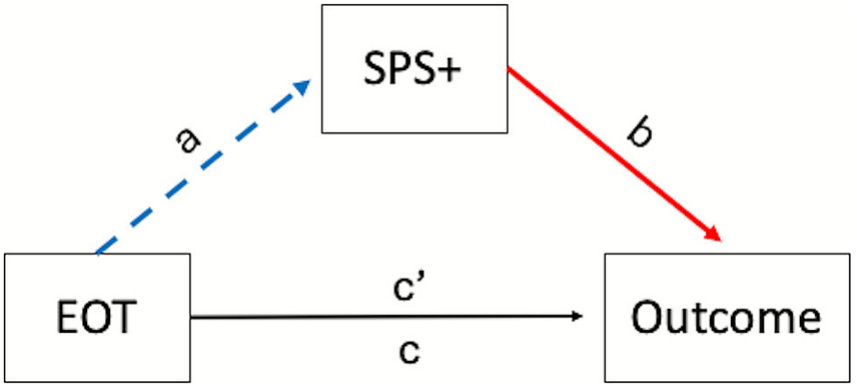
General form of the models assessing mediation by positive traits associated with sensory processing sensitivity (SPS+) in Studies 1 and 2. The dashed blue line denotes a negative relationship, and the solid red line denotes a positive relationship; the black line denotes a non-significant path. The predictor in all cases was the score on the externally oriented thinking (EOT) subscale of the Toronto Alexithymia Scale. The outcome variable for Study 1 was the total score on the Interoceptive Accuracy Scale; outcomes for the three mediations reported in Study 2 included the Noticing, Attention Regulation, and Emotional Awareness subscale scores from the Multidimensional Assessment of Interoceptive Awareness.

To test whether SPS+ traits mediated the relationship between EOT and interoceptive accuracy we conducted a mediation analysis using Hayes’ PROCESS model 4. DIF and DDF were not included in the model given our small sample size and the fact that neither variable was significantly correlated with the mediator or outcome variable. In this and other mediation analyses described below, tests of the significance of indirect effects relied on examination of percentile bootstrap 95% confidence intervals (CIs) generated from 5,000 bootstrap samples. Full mediation was supported (indirect effect: ab = −0.178, boot 95%CI [−0.352, −0.039]; see Figure 1). Thus, those who failed to turn their attention inward (high EOT) also reported being less sensitive to subtle internal and external cues (low SPS+; a = −0.051 [−0.074, −0.028]), and this predicted lower subjective interoceptive accuracy (low IAS; b = 3.481[0.931, 6.032]). These results provide preliminary support for the proposed indirect relationship between EOT and subjective interoceptive accuracy.

### 2.3 Discussion

The present findings draw into question the idea that interoceptive propensity and interoceptive accuracy are distinct (e.g., Murphy et al., 2020). We suggest instead that the relationship between these two dimensions of interoception is indirect. Although causal inferences cannot be drawn from our study due to its cross-sectional design, the findings are consistent with the idea that those who score high on EOT may be less aware of how subtle internal (and external) stimuli resonate in their bodies due to their weak interoceptive propensity, and that this leads them to feel less able to perceive these cues accurately. If this can be confirmed in future experimental work, it might suggest that the negative association that has been reported between *total* scores on the TAS-20 and the IAS (Murphy et al., 2020) reflects, in part, the fact that individuals with the highest TAS-20 scores report strong EOT in addition to strong DIF and DDF. Our results speak to the importance of examining how the specific subscales of the TAS-20 relate to different facets of subjective interoception, rather than relying on a global measure such as the TAS-20 total score. They also suggest that it would be prudent for researchers interested in studying links between alexithymia and subjective interoception to include measures of the positive and negative facets of SPS in their study protocols.

We argued earlier that there are certain advantages to studying subjective measures of interoception, but we acknowledge that scores on the IAS may not reliably reflect true physiological sensitivity or detection ability. For this reason, incorporating an objective measure of interoceptive accuracy would be important in a future study, particularly as Trevisan et al. (2021) found a weak association between global alexithymia scores and objective measures of interoceptive accuracy. We predict that individuals with a strong interoceptive propensity (low EOT), who tend to score high on SPS+ traits, may find it relatively easy to monitor subtle internal cues accurately—especially if attending to these cues further amplifies their salience (Jakobson and Rigby, 2021) and/or leads to more frequent updating of prediction errors (see Ainley et al., 2016; Hübner et al., 2021). If so, they should perform well when trying to monitor subtle signals in objective testing. If the signals are strong or unpleasant, however, it is possible that those who score high on DIF or DDF, who tend to strongly express SPS– traits and are hypersensitive to this type of stimulation (Jakobson et al., 2024), may frequently try to distract themselves in an effort to manage their arousal, and that this tendency would be linked to worse performance in objective testing. Importantly, when testing these hypotheses, it would be prudent to measure interoceptive accuracy in several objective domains (e.g., cardiac, gastroesophageal), as these relationships may be more evident in some systems than others (Desmedt et al., 2023b).

The main goal of Study 2 was to explore the possibility that SPS+ traits might mediate links between EOT and aspects of “adaptive interoception” (Desmedt et al., 2022) measured by the MAIA-2. We also explored possible links between DIF/DDF, SPS– traits, and dimensions of subjective interoception that may be maladaptive, including the tendencies to worry about and try to distract oneself from unpleasant forms of stimulation.

## 3 Study 2

As noted earlier, subjective measures of interoception vary widely in the types of phenomena they tap into. Using both factor and network analysis, Desmedt et al. (2022) found support for grouping items from some of the more widely used self-report measures into different clusters. Items that fell in their “*extero-interoceptive awareness*” cluster and in their “*adaptive interoception”* cluster (the latter including items from select subscales of the MAIA-2) appear to measure features that overlap considerably with the SPS+ trait cluster (Jakobson et al., 2024). As such, we might expect them to be directly or indirectly linked to low scores on EOT. In contrast, Desmedt et al. (2022) described other clusters of items that seemed to tap into overlapping and potentially maladaptive processes. These included a “*negative feelings propensity*” cluster, a “*neutral and negative body sensations awareness*” cluster, and a cluster that included two MAIA-2 subscales—low scores on which measure the tendencies to worry about sensations of pain or discomfort and to try to distract from them (e.g., by using avoidance coping). It seems likely that scores on these clusters of items would be more strongly associated with SPS– traits. If so, we might expect scores on these measures to be directly or indirectly linked to DIF and/or DDF.

The goal of Study 2 was to provide a preliminary test of the hypothesis that different SPS trait clusters would mediate links between specific facets of alexithymia and aspects of interoception captured by the MAIA-2 (Mehling et al., 2018). Given the results of Study 1, we expected to find that SPS+ traits would negatively mediate the relationship between EOT and many of the adaptive constructs sampled by the MAIA-2—particularly those relating to attention regulation and the tendency to notice bodily sensations, and the tendencies to link bodily sensations to emotional states and listen to the body for insight. Indeed, a recent meta-analysis suggested a small, negative relationship between EOT and all MAIA subscales, except for Not Worrying (Van Bael et al., 2024). This meta-analysis also highlighted small, negative relationships between DIF/DDF and all MAIA subscales. In the current work, however, we expected to find that DIF/DDF would be most strongly related to low scores on the NW and ND subscales, and that these relationships would be mediated by SPS– traits.

### 3.1 Materials and methods

#### 3.1.1 Participants

Participants were recruited through convenience sampling from students enrolled in an Introduction to Psychology course, who earned credit toward a research participation option. After exclusions (see below), the final sample included 98 individuals (49 females, *M*_age_ = 20.9 years, *SD* = 6.4). The sample size was expected to exceed that needed to detect a medium effect with power set at 0.80 in the multiple regressions we conducted (as determined using G*Power 3.1.9.7). It was also expected to provide adequate power to test our planned mediation models based on both the standard rule-of-thumb (as there were 33 cases per parameter) and the minimum sample size needed to detect full simple mediation with medium effect size suggested by Sim et al. (2022). Unless otherwise indicated, a post-hoc analyses confirmed that the observed power for the mediation models tested was acceptable, ranging from 0.77 to 0.99. The protocol was approved by our institutional Research Ethics Board and all participants provided informed consent.

#### 3.1.2 Procedure and measures

After providing informed consent and completing a commitment request item, participants provided information about their biological sex (*Male, Female,* or *Prefer not to say*) and age. They then completed the TAS-20, HSPS, MAIA-2, and OS questionnaires in that order, with the five items comprising the CRS being randomly distributed across the questionnaires. The survey was mounted on the Qualtrics survey platform.

See Study 1 for descriptions of the TAS-20, HSPS, OS and CRS. We computed SPS+ and SPS– composite scores as described in Study 1. Cronbach’s alphas for the TAS-20, the SPS+, and the SPS– items in the present study were 0.84, 0.72, and 0.85, respectively. Measures unique to the present study are described below.

##### 3.1.2.1 Commitment request

In addition to completing the CRS, participants were asked to indicate if they would commit to providing thoughtful answers to the questions in the survey. Response options were “Yes, I will,” “No, I will not,” and “I cannot promise either way.”

##### 3.1.2.2 Multidimensional Assessment of Interoceptive Awareness

The MAIA-2 (Mehling et al., 2018) is a 37-item self-report measure tapping into a range of constructs related to interoception. Individuals indicate the degree to which each statement applies to them using a 6-point Likert scale (from 0 = *Never* to 5 = *Always*). After reverse-scoring, where indicated, eight subscale scores are derived by averaging responses to relevant items. Based on Desmedt et al.’s (2022) findings, the “adaptive interoception” subscales include: Noticing (N; being aware of uncomfortable, comfortable, and neutral body sensations, 4 items), Attention Regulation (AR; the ability to sustain and control attention to body sensations, 7 items), Emotional Awareness (EA; awareness of the connection between body sensations and emotional states, 5 items), Self-Regulation (SR; the ability to regulate distress by attending to body sensations, 4 items), Body Listening (BL; actively listening to the body for insight, 3 items), and Trusting (T; experiencing one’s body as safe and trustworthy, 3 items). As noted above, the remaining two subscales, ND (not ignoring or avoiding sensations of pain or discomfort, 6 items) and NW (not worrying or experiencing distress with sensations of pain or discomfort, 5 items), formed separate clusters in Desmedt et al.’s (2022) analysis and we considered them separately. Cronbach alphas for the subscales of the MAIA-2 in the present sample ranged from 0.67 to 0.87.

#### 3.1.3 Data cleaning

A total of 107 individuals took part in the study. Two individuals were excluded for failing to commit to providing thoughtful answers to the questions in the survey, three more were excluded for failing the attention check, and four were excluded for failing to answer the majority of items comprising one or more of the questionnaires, leaving a final sample of 98.

There were 1.22% missing data on the OS. Little’s Missing Completely at Random test (Li, 2013) confirmed that data were missing completely at random and expectation maximization was used to estimate missing data points. All analyses were carried out using SPSS v.29.

### 3.2 Results

Descriptive statistics and zero-order correlations between study variables are provided for the interested reader in Table 2. Although it was not our primary interest here, to facilitate comparisons across the present studies and between our data and previous reports, we draw the reader’s attention to the correlations between TAS-20 total scores and scores on the MAIA-2 subscales. After Benjamini-Hochberg correction for multiple comparisons, *p* values for three of these eight correlations were significant. High scores on the TAS-20 were associated with low scores on AR (*p* = 0.008), SR (*p* = 0.045), and T (*p* = 0.028). A relationship between high TAS-20 total scores and low scores on NW approached significance (*p* = 0.068).

**Table 2:** Study 2: descriptive statistics and zero-order correlations between study variables.

Measure	*M*	*SD*	TAS	DIF	DDF	EOT	SPS+	SPS–	N	AR	EA	BL	SR	T	ND
TAS	53.1	11.6	--												
DIF	18.0	5.8	0.889***	--											
DDF	15.3	4.4	0.825***	0.652***	--										
EOT	19.7	4.1	0.678***	0.392***	0.333***	--									
SPS+	4.9	0.6	−0.200*	**−0.109**	**−0.089**	−0.314**	--								
SPS–	4.2	1.0	0.371***	0.463***	0.305**	**0.064**	0.151	--							
N	3.1	0.8	−0.088	−0.085	−0.068	−0.054	0.312**	**0.172**	--						
AR	2.9	0.7	−0.368**	−0.308**	−0.312**	−0.268**	0.362***	**−0.122**	0.284**	--					
EA	3.3	0.8	−0.002	0.070	0.015	−0.120	0.403***	**0.160**	0.410***	0.076	--				
BL	2.5	1.0	−0.074	−0.063	−0.116	0.003	**0.099**	**0.139**	0.413***	0.035	0.436***	--			
SR	2.7	1.1	−0.240*	−0.280**	−0.215*	−0.051	**0.150**	**−0.175**	0.427***	0.345***	0.358***	0.511***	--		
T	3.5	1.0	−0.270**	−0.308**	−0.147	−0.168	**0.141**	**−0.164**	0.215*	0.394***	0.133	0.209*	0.385***	--	
ND	1.8	0.8	−0.154	−0.230*	−0.167	0.072	**−0.174**	−0.236*	0.000	−0.200*	−0.022	0.187	0.203*	0.036	--
NW	2.4	0.8	−0.214*	−0.189	−0.207*	−0.115	**−0.087**	−0.486***	−0.119	0.207*	−0.029	−0.110	0.127	0.150	−0.087

*N* = 98. Correlations of particular interest are in bold. Toronto Alexithymia Scale total score (TAS) and subscales: difficulty identifying feelings (DIF); difficulty describing feelings (DDF); externally oriented thinking (EOT). Positive (SPS+) and negative (SPS–) facets of sensory processing sensitivity. Subscales of the Multidimensional Assessment of Interoceptive Awareness-2 (MAIA-2): Noticing (N), Attention Regulation (AR), Emotional Awareness (EA), Body Listening (BL), Self-regulation (SR), Trusting (T), Not Distracting (ND), and Not Worrying (NW). *p < 0.05 without correction for multiple comparisons. **p < 0.01 without correction for multiple comparisons. ***p < 0.001 without correction for multiple comparisons.

Our main goal was to explore links between facets of alexithymia, SPS, and interoception. As such, 22 of the zero-order correlations (those shown in bold in the table) were of particular interest. With one exception noted below, all significant correlations discussed had *p* values ≤ 0.006 after Benjamini-Hochberg correction for multiple comparisons.

Consistent with our expectations we found that: (a) EOT was negatively correlated with SPS+ (but not SPS–); and (b) SPS+ (but not SPS–) was positively correlated with N, AR, and EA. On these grounds, we proceeded to test the hypotheses that SPS+ traits would negatively mediate the relationship between EOT and scores on each of these three MAIA-2 subscales. The analyses were run using model 4 of Hayes’ PROCESS macro. SPS+ traits fully negatively mediated the relationship between EOT and AR (indirect effect = −0.017, boot 95%CI [−0.038, −0.003]), N (indirect effect = −0.020, boot 95%CI [−0.038, −0.005]), and EA (indirect effect = −0.026, boot 95%CI [−0.047, −0.008]). Thus, people who had a strong external focus (high EOT) also reported being less sensitive to subtle signals (low SPS+; a = −0.046 [−0.074, −0.018]) and this tendency, in turn, was linked to a reduced capacity to sustain and control attention to body sensations (low AR; b = 0.381 [0.138, 0.624]) along with a reduced awareness of these sensations (low N; b = 0.440 [0.166, 0.713]) and their links to emotions (low EA; b = 0.573 [0.295, 0.852]).

We had expected to see a negative association between EOT and BL and that this link might also be mediated by SPS+. However, we did not test this directly because SPS+ scores were not correlated with BL in our sample, making testing for mediation unwarranted. The same was true of SR and T; however, visual inspection of Table 2 suggested that both of these measures might be more closely linked to DIF than to EOT. Although we had not anticipated this, we felt it would be useful to determine if this was the case, as it might guide future investigations. As such, we report here the findings from two, exploratory multiple regression analyses in which the three facets of alexithymia were entered as predictors. Forced entry was used and coefficients were estimated using bias corrected and accelerated 95% confidence intervals (BCa 95% CI) based on 1,000 bootstrap samples. Both models were significant (*β* ≤ −0.267, *R*^2^ ≥ 0.085, *F*(3,94) ≥ 2.912, *p* ≤ 0.038 in both cases). Individuals who endorsed having difficulty distinguishing feelings from other bodily states (high DIF) were more likely to report finding it difficult to regulate distress by attending to body sensations (low SR) and to report not viewing their bodies as safe and trustworthy (low T). It is unlikely that these relationships were mediated by SPS– because neither SR nor T was significantly correlated with SPS– composite scores.

**Figure 2:**
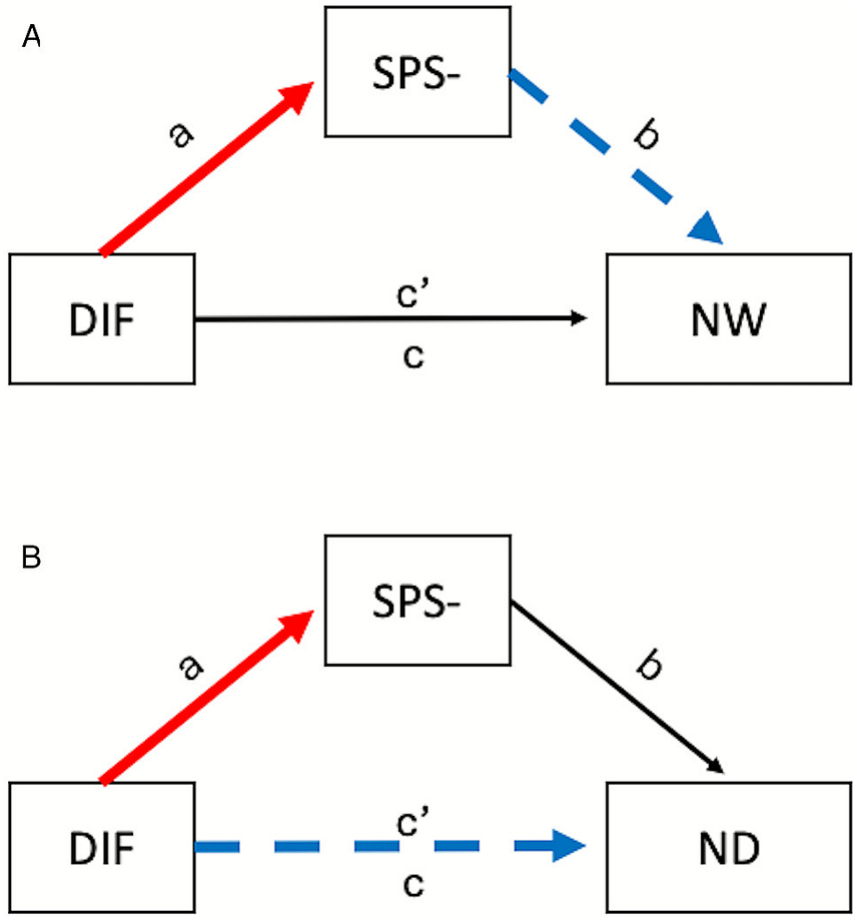
General form of the models assessing mediation by negative traits associated with sensory processing sensitivity (SPS–) in Study 2. The dashed blue lines denote a negative relationship, while the solid red lines denote a positive relationship; the black line denotes a non-significant path. The predictor in all cases was the score on the difficulty identifying feelings (DIF) subscale of the Toronto Alexithymia Scale. The outcome variables were the not worrying (NW; **A**) and not distracting (ND; **B**) subscales of scores from the Multidimensional Assessment of Interoceptive Awareness.

As expected: (a) DIF and DDF (but not EOT) were positively correlated with SPS– traits; and (b) stronger expression of SPS– (but not SPS+) traits was correlated with the tendencies to worry about sensations of pain or discomfort (low NW) and to try to distract oneself from such sensations (low ND), although the latter effect was only marginally significant (*p* = 0.052) after Benjamini-Hochberg correction. As DIF showed a stronger relationship to SPS– traits than DDF (see Table 2), we tested for positive mediation by SPS– of the link between DIF and scores on these two MAIA-2 subscales. The relationship between DIF and NW was fully mediated by SPS– traits (indirect effect = −0.033, boot 95%CI [−0.055, −0.017]; see Figure 2A). Thus, those scoring high on DIF also reported being more sensitive to uncomfortable forms of stimulation (high SPS–; *a* = 0.076 [0.046, 0.105]) and this predicted reporting a heightened tendency to worry about or become emotionally distressed by uncomfortable sensations (low NW; *b* = −0.439 [−0.613, −0.265]) Mediation was not supported in the model predicting scores on the ND subscale (see Figure 2B); however, the total effect (the sum of direct and indirect effects) was significant (*c*’ = −0.034 [−0.062, −0.005]), providing provisional support for the view that DIF may be associated with a tendency to try to ignore or distract oneself from sensations of pain or discomfort (low ND). We urge caution in interpreting this result, however, as the observed power for this mediation analysis was unacceptably low.

### 3.3 Discussion

Our observation that total scores on the TAS-20 were negatively correlated with scores on several MAIA-2 subscales is consistent with other findings (e.g., Van Bael et al., 2024; Desdentado et al., 2023; Zahid et al., 2024) and complements past reports linking this global measure of alexithymia to interoceptive impairment more broadly (Murphy et al., 2018). The present results extend this work by providing preliminary evidence that components of SPS mediate the relationships between specific facets of alexithymia and particular dimensions of subjective interoception. Before discussing our findings in detail, we want to highlight the fact that our facet-level approach differs from that taken by many others. When running exploratory regressions we included all three TAS-20 subscales as predictors. Before running mediation models, we checked to see if facets of alexithymia other than the one serving as the predictor were associated with the mediator and/or the outcome variable. These steps were important because DIF, DDF, and EOT are intercorrelated—making it challenging to isolate variance in outcome measures attributable to a specific facet. A stronger test for mediation would be to include all three facets of alexithymia as predictors (and both facets of SPS as mediators) in each model; however, our sample size did not provide adequate power to do this. As such, the findings presented here should be interpreted cautiously.

People who reported having a strong external focus (high EOT) also tended to be less sensitive to subtle stimuli (low SPS+) and this, in turn, appeared to be related to a reduced capacity to notice and to sustain/control attention to body sensations, and to connect these sensations to emotional states. In contrast to other groups (e.g., Van Bael et al., 2024; Zahid et al., 2024; Zamariola et al., 2018), we did not establish a link between EOT and BL in this study; however, it is possible that EOT might exert a downstream effect on this facet of interoception as well. Interestingly, other work has shown that low scores on N, AR, EA, and BL are all associated with low scores on the IAS (Brand et al., 2023). This lends support to the suggestion we made in the Discussion of Study 1 that, whereas individuals scoring high in EOT may be less likely to notice when they are having difficulties, individuals scoring low in EOT (who tend to be more sensitive to subtle cues) may be able to use top-down attentional control to amplify weak signals in order to facilitate monitoring. This could be quite adaptive from an evolutionary perspective (e.g., it might prompt one to rest when one’s heart rate increases due to heavy exertion), but not if taken to an extreme.

The present findings also add to the literature examining links between alexithymia, potentially maladaptive aspects of interoception, and emotion dysregulation (e.g., Desdentado et al., 2023; Li et al., 2024). First, we found that DIF was associated with a heightened tendency to worry about or become emotionally distressed by uncomfortable sensations via its association with strong SPS– traits. Second, we found that DIF was linked to the tendency to try to ignore or distract oneself from uncomfortable sensations. While this coping strategy can be effective in the short term or when combined with active acceptance, the habitual or excessive use of distraction and avoidance to cope with distress is generally considered maladaptive from a psychological perspective (e.g., Wolgast and Lundh, 2017). Finally, we found that—when controlling for DDF and EOT—DIF emerged as a significant predictor of finding it difficult to regulate distress by attending to body sensations (low SR) and of the tendency to not view one’s body as safe and trustworthy (low T). It will be important in future work to explore factors that may underpin each of these relationships, as this may provide new insights into psychological processes that increase risk for common mental health problems such as depression (Li et al., 2024).

Being hypervigilant to subtle, internal signals and using maladaptive coping strategies to combat unpleasant sensations also likely contribute to anxiety sensitivity, which is characterized by having an unrealistic fear of anxiety-related sensations and their possible negative consequences (Reiss et al., 1986). Van Landeghem and Jakobson (2024) have shown that anxiety sensitivity, alexithymia, and SPS frequently co-occur, but they also found that each of these variables accounts for unique variance in anxiety and depression symptoms. More research is needed to assess the individual contributions that these variables make to other conditions, such as somatic symptom disorder and posttraumatic stress disorder. We encourage future researchers to adopt a facet-level approach in this work and that, as suggested by Desmedt et al. (2023b), they test whether relationships hold across domains of interoception (e.g., cardiac, gastroesophageal) or are more evident in some systems than others.

## 4 Study 3

Interoception is widely believed to play a key role in our ability to represent not only our own inner states and subjective feelings, but also those of other people (e.g., Hübner et al., 2021). An important cue to another’s emotional state is, of course, their facial expression. Prochazkova and Kret (2017) have suggested that seeing another person’s expression leads to the production of a prediction model (via shared neural activation) and that we use automatic mimicry of the other’s movements (e.g., facial mimicry, gaze synchrony) and their inner state (e.g., heart rate synchronization, pupil mimicry, blushing) to reduce prediction errors. The product of these two forms of automatic mimicry, which arises through multisensory integration, is emotional contagion. As noted earlier, it has been suggested that people with alexithymia may experience problems with multisensory integration that disrupt predictive coding and that, if this happens, it can lead them to adopt an externally focused thinking style (see Pollatos and Herbert, 2018). We propose that by *routinely* directing their attention outward (i.e., by developing a low interoceptive propensity) the difficulties that alexithymic individuals who score high on EOT may have in integrating external and internal cues could become amplified. If this occurs, it may impact emotional contagion and contribute not only to atypicalities in how others’ emotions are represented and/or interpreted, but also to the establishment of biases in social perception—as reflected in things such as ratings of how approachable or trustworthy others are judged to be (e.g., Willis et al., 2011a,b).

The key objective of Study 3 was to determine if aspects of subjective interoception captured by the MAIA-2 might mediate links between EOT and perceptual and/or social judgments of others. Participants completed a behavioral task in which they were required to make judgments of the intensity and valence (pleasantness) of others’ facial expressions, and about how likely they would be to approach each person to ask for assistance. As each stimulus face was rated on all three scales, the working memory and sustained attention demands of the task were comparable across the three ratings. We were especially interested in studying faces displaying expressions that were nuanced (i.e., not strongly negatively or positively valenced), as emotional contagion is thought to be particularly important for decoding subtle or ambiguous expressions and when making difficult judgments (Wood et al., 2016). Although we did not collect measures of SPS in this study it would be interesting to do so in a future study given that SPS+ traits include sensitivity to subtle and interpersonal cues (De Gucht et al., 2022). Indeed, it has been reported elsewhere that sensitivity to subtle stimuli predicts the strength of the mixed emotional response elicited by seeing someone in an embarrassing situation, as well as the extent to which one believes their own reaction matches that of the person being viewed (McQuarrie et al., 2023). We expected to find that EOT would be related to adaptive aspects of interoception sampled by the MAIA-2 such as AR and that these variables, in turn, would predict variability in perceptual and social judgments regarding faces displaying nuanced expressions.

### 4.1 Materials and methods

#### 4.1.1 Participants

Participants were recruited via convenience sampling through an Introduction to Psychology participant pool and were awarded credit toward a research participation option for taking part. After exclusions (see below), the final sample included 53 individuals (26 female, *M*_age_ = 19.8 years, *SD* = 3.9). The sample size was expected to exceed that needed to detect a medium effect with power set at 0.80 in our regressions (as determined using G*Power 3.1.9.7). The protocol was approved by our institutional Research Ethics Board and all participants provided informed consent.

#### 4.1.2 Procedures and measures

Participants completed the study in a computer laboratory in groups of 15 to 20 people. The room was set up to allow each participant to work independently at a computer station. Participants completed the TAS-20 (described in Study 1) and the MAIA-2 (described in Study 2) via the Qualtrics survey platform (https://www.qualtrics.com). Items from the CRS (described in Study 1) were randomly interspersed amongst items from these measures. After completing the questionnaires, they went on to complete the perceptual task, which was programmed using E-prime 3.0 software (Psychology Software Tools, Pittsburgh, PA).

Face stimuli used in the perceptual task were obtained with permission from Cordaro et al. (2019). Their face database includes photographs of actors expressing 18 different psychological states, including: disgust, anger, fear, sadness, pain, boredom, shame, desire, confusion, sympathy, pride, surprise, coyness, embarrassment, interest, amusement, contentment, and happiness. Past research using this stimulus set suggests that participants from nine diverse cultures could reliably identify all expressions (Cordaro et al., 2019); however, as can be seen in the supplementary materials that accompany that article, some were recognized more accurately than others. For example, in the American sample (which would presumably be most similar to our local sample), mean recognition rates for the full set of stimuli ranged from 56.4 to 95.5%, suggesting that some of the expressions depicted were nuanced and more difficult to identify. From each of the 18 expression categories in the full Cordaro et al. (2019) database, we selected photographs of two European American actors (one male and one female actor). We used data collected in our perceptual task (described below) to identify the expressions in this restricted set of stimuli that were most nuanced and, therefore, of greatest interest.

The perceptual task was run in three blocks, with each of the 36 stimulus faces being shown once per block. In each trial of a given block, a stimulus face (13.5 cm wide and 11 cm high) appeared on the computer screen along with a question and response options. In the valence-rating block, participants responded to the question “How pleasant is this expression?” using a scale ranging from −4 (*very unpleasant*) to 0 (*neutral*) to +4 (*very pleasant*). In the intensity-rating block, participants responded to the question “How intense is this expression?” using a scale ranging from −4 (*very low in intensity*) to 0 (*moderate in intensity*) to +4 (*very high in intensity*). In the approachability-rating block, they responded to the question “How likely would you be to approach this person to ask for directions?” using a scale ranging from −4 (*definitely avoid*) to 0 (*I do not know*) to +4 (*definitely approach*). The stimulus, statement, and response options remained on the screen until the participant responded via a mouse click. Viewing distance during the perceptual task was approximately 50 cm.

The E-Prime program randomized trial order within a block and counterbalanced the order of the blocks so that half of the participants completed valence ratings first, followed by intensity ratings, and then approachability ratings; and the other half of the participants made approachability ratings first, followed by valence, and then intensity ratings.

#### 4.1.3 Data cleaning and processing

The protocol was completed by 58 individuals. We excluded individuals who scored low on the Conscientious Responders Scale (*n* = 2), those who had extreme scores (±3 SD from the mean) on one or more MAIA-2 subscales (*n* = 1), and those who (due to technical difficulties) did not complete all blocks of the perceptual task (*n* = 2), leaving a final sample of *N* = 53. There were no missing data in the questionnaire responses. We used the mean rating derived from available data for the relevant stimulus face to estimate the small amount of missing data in the perceptual task. All multiple regression models were run using the forced entry method, and coefficients were estimated using BCa 95% CIs based on 1,000 bootstrap samples. All statistical analyses were carried out using SPSS (v 29).

### 4.2 Results

Descriptive statistics and zero-order correlations between the study variables are provided for the interested reader in Table 3.

**Table 3:** Study 3: descriptive statistics and zero-order correlations between study variables.

Measure	*M*	*SD*	TAS	DIF	DDF	EOT	N	AR	EA	BL	SR	T	ND	NW	INT	PLE
TAS	3.2	0.9	0.852***	--												
DIF	2.9	1.0	0.768***	0.543***	--											
DDF	3.2	1.0	0.660***	0.291*	0.288*	--										
EOT	2.6	1.0	−0.212	−0.154	−0.245	−0.101	--									
N	2.1	1.2	−0.387**	−0.307*	−0.236	−0.341*	0.593***	--								
AR	3.5	1.1	−0.230	−0.175	−0.143	−0.211	0.502***	0.498***	--							
EA	1.9	0.9	−0.200	0.026	−0.249	−0.312*	0.212	0.250	0.451***	--						
BL	2.6	2.6	−0.339*	−0.187	−0.285*	−0.341*	0.253	0.504***	0.373**	0.420**	--					
SR	47.1	10.6	−0.237	−0.287*	−0.151	−0.070	0.196	0.417**	0.283*	0.188	0.439***	--				
T	13.4	3.8	−0.128	−0.097	−0.125	−0.076	0.212	−0.131	−0.019	0.018	−0.143	−0.035	--			
ND	15.3	5.7	0.089	0.030	0.009	0.173	−0.263	−0.048	−0.133	−0.094	−0.067	0.006	−0.302*	--		
NW	18.5	4.2	−0.093	−0.040	−0.196	−0.002	0.349*	0.393**	−0.013	0.063	0.130	−0.018	0.009	0.020	--	
INT	0.1	0.9	−0.093	−0.040	−0.196	−0.002	0.349*	0.393**	−0.013	**0.063**	0.130	−0.018	0.009	0.020	--	
PLE	−0.4	0.6	−0.020	−0.046	0.104	−0.081	0.010	**0.014**	−0.130	−0.323*	0.087	0.069	−0.237	−0.059	−0.05	--
APP	−0.1	1.1	0.017	−0.031	0.020	0.066	0.048	**−0.006**	−0.178	−0.286*	−0.035	0.090	−0.068	0.051	−0.003	0.654***

*N* = 53. Correlations of particular interest are in bold. Toronto Alexithymia Scale total score (TAS) and subscales: DIF = difficulty identifying feelings; DDF = difficulty describing feelings; EOT = externally oriented thinking. Subscales of the Multidimensional Assessment of Interoceptive Awareness (MAIA-2): N = Noticing, AR = Attention Regulation, EA = Emotional Awareness, BL = Body Listening, SR = Self-Regulation, T = Trusting, ND = Not Distracting, NW = Not Worrying. Ratings from the perceptual task: INT = intensity; PLE = pleasantness; APP = approach. *p < 0.05 without correction for multiple comparisons. **p < 0.01 without correction for multiple comparisons. ***p < 0.001 without correction for multiple comparisons.

#### 4.2.1 Identifying the nuanced expressions

Because we were using only a subset of the Cordaro et al. (2019) stimuli, we could not rely on their data to identify the subset of expressions that were nuanced, as mean ratings for their sample were not provided for individual stimuli. As a result, before we could move on to test our hypotheses, we needed to perform some preliminary analyses to identify our target stimuli. By definition, nuanced expressions are often not clearly positively or negatively valenced and can be quite subtle or hard to interpret. We also expected them to generate more uncertainty regarding how approachable the individual depicted was likely to be, given that they should, in theory, generate weaker or more diffuse emotional contagion than more strongly valenced expressions.

Given the above, as an initial step in identifying our target expressions, we computed the mean pleasantness rating for each expression. These mean ratings were then rank-ordered and the expressions were assigned to one of three categories: ones viewers thought were unpleasant (mean rating < −1: disgust, anger, bored, sadness, pain, fear), ones viewers thought were pleasant (mean rating > +1: coy, embarrassed, content, amused, happy), and ones that appeared to be more nuanced (i.e., not clearly positively or negatively valenced; mean rating between −1 and +1: shame, confusion, sympathy, desire, pride, surprise, interest).

Next, we tested a linear mixed-effects model to determine if the expressions in our nuanced category were also rated as being less intense than expressions in the other categories. The fixed effects of category were statistically significant, *F*(2,104) = 32.723, *p* < 0.001. For random effects, the variance component for participant was 0.325 (SE 0.138). Pairwise comparisons confirmed that the nuanced expressions (*M* = 0.124, 95% CI [−0.190, 0.438]) were rated as being less intense than the unpleasant expressions (*M* = 1.47, 95% CI [1.152, 1.780]; *p* < 0.001) but were of similar intensity to the pleasant expressions (*M* = 0.072, 95% CI [−0.242, 0.386]).

Finally, we tested a linear mixed-effects model to determine if, as expected, participants would report being less sure about whether to approach or avoid people displaying the expressions we had tentatively assigned to the nuanced category. The fixed effect of category was again statistically significant, *F*(2,104) = 287.082, *p* < 0.001. For random effects, the variance component for participant was 0.690 (SE 0.170). Pairwise comparisons confirmed that viewers felt they should avoid the people with unpleasant expressions (*M* = −1.537, 95% CI [−1.841, −1.233]) and were willing to approach those with pleasant expressions (*M* = 1.900, 95% CI [1.596, 2.204]), but that they were unsure whether to avoid or approach those displaying nuanced expressions (*M* = −0.148, 95% CI [−0.451, 0.156]) (*p* < 0.001 for all contrasts). Thus, our expectations were fully supported by this analysis.

#### 4.2.2 Links between EOT and interoception, and between interoception and perceptual/social judgments regarding faces displaying nuanced expressions

Although it was not our primary interest here, to once again facilitate comparisons across studies and between our data and previous reports, we draw the reader’s attention to the set of eight correlations between TAS-20 total scores and scores on the MAIA-2 subscales. After Benjamini-Hochberg correction for multiple comparisons, high TAS-20 total scores were found to be significantly correlated with low scores on the AR subscale (*p* = 0.032), and a similar pattern was evident with scores on the SR subscale (*p* = 0.052).

As a first step in addressing the main goal of our study, we sought to determine which scales of the MAIA-2 were most closely associated with EOT when controlling for DIF and DDF. To do this, we ran separate multiple regressions predicting scores on each of the MAIA-2 subscales. In our relatively small sample, only the models predicting AR [*R*^2^ = 0.165, *F*(3,49) = 3.234, *p* = 0.030], SR [*R*^2^ = 0.154, *F*(3,49) = 2.984, *p* = 0.040], and BL [*R*^2^ = 0.181, *F*(3,49) = 3.616, *p* = 0.019] were significant. Based on inspection of the BCa 95% CIs, none of the three TAS-20 subscales accounted for significant variance in SR on their own. However, AR was negatively predicted by EOT (*b* = −0.062 [−0.122, −0.002]), and BL was negatively predicted by EOT (*b* = −0.084 [−0.149, −0.007]) and positively predicted by DIF (*b* = 0.058 [0.010, 0.102]). Failure to replicate other links found in Study 2 likely reflects limited statistical power.

Our next step was to determine if individual differences in either AR or BL were associated with viewers’ ratings of the intensity or pleasantness of nuanced expressions, or with their ratings of the approachability of the people displaying these expressions. As can be seen in Table 3, AR was positively related to ratings of expression intensity (adjusted *p* = 0.028), and BL was negatively related to both pleasantness ratings and approach motivation (although adjusted *p* values were only marginally significant, *p* ≤ 0.076).

#### 4.2.3 Exploratory mediation analyses

Based on the findings presented above, we performed two tests of formal mediation. The first assessed the possibility that AR might negatively mediate an association between EOT and ratings of the intensity of nuanced expressions. The second assessed the possibility that BL might positively mediate an association between EOT and ratings of the pleasantness of nuanced expressions and/or approach motivation. As past research suggests that the valence of a facial expression is a strong driver of perceived approachability (Willis et al., 2011a) this second analysis involved a test for serial mediation. Although we had 18 cases per parameter (meeting the standard rule-of-thumb guidelines), the sample size was lower than the minimum needed to detect full simple mediation with medium effect size (Sim et al., 2022) or full serial mediation (as per https://schoemanna.shinyapps.io/mc_power_med/). Indeed, post-hoc analyses confirmed that the observed power for the significant mediations described below was unacceptably low (0.48 to 0.64). Nonetheless, we report the findings here as these exploratory analyses may inform future research aimed at testing formal mediation in larger samples.

**Figure 3:**
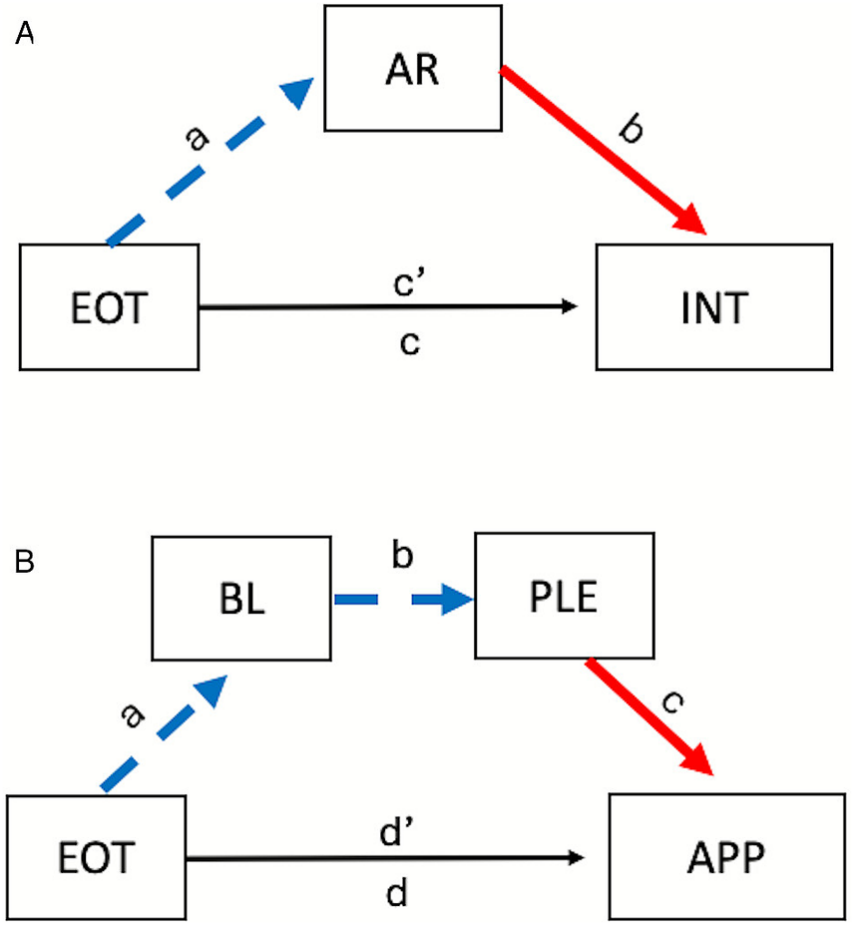
General form of the models assessing simple **(A)** and serial **(B)** mediation of the links between externally oriented thinking (EOT) and perceptual and social judgments made in Study 3. The dashed blue line denotes a negative relationship, while the solid red line denotes a positive relationship; the black line denotes a non-significant path. AR = Attention regulation; BL = Body listening; INT = intensity; PLE = pleasantness; APP = approachability.

The first analysis, designed to test the hypothesis that AR might negatively mediate the link between EOT and intensity ratings, was carried out using PROCESS model 4. Full mediation was supported (indirect effect = −0.032, boot CI [−0.060, −0.003]). As shown in Figure 3A, the tendency to maintain an external focus (high EOT) was linked to a diminished ability to sustain and control attention to body sensations (low AR: *a* = −0.079 [−0.140, −0.018]), which predicted lower ratings of the intensity of nuanced expressions (*b* = 0.405 [0.155, 0.656]).

In our second analysis, we used PROCESS model 6 to test for serial mediation; EOT was entered as the predictor, BL as the first mediator, the pleasantness rating as the second mediator, and approach motivation as the outcome. Full serial mediation was supported (indirect effect = 0.023, boot CI [0.003, 0.053]). As shown in Figure 3B, high EOT predicted low BL scores (*a* = −0.096 [−0.173, −0.019]), which predicted finding nuanced expressions more pleasant (*b* = −0.210 [−0.363, −0.058]), which predicted a greater willingness to approach the people displaying these expressions for help (*c* = 1.121 [0.718, 1.523]).

### 4.3 Discussion

The present results support the view that EOT is related to problems in facets of interoception that may impact emotional contagion and embodied cognition. Extending prior work (Van Bael et al., 2024) we showed that EOT predicted reduced AR and BL after controlling for DIF and DDF, but we went on to show that: (a) lower AR predicted lower ratings of the intensity of nuanced expressions, and (b) that having a weaker tendency to listen to one’s body for insight predicted finding these expressions more pleasant, which predicted finding the people displaying these expressions more approachable. Although we did not measure SPS here, we suspect that SPS+ may play an important role in these effects, given that self-reported sensitivity to subtle cues (regardless of valence) and to social affective cues are key characteristics of those reporting strong SPS+ traits (De Gucht et al., 2022).

Our finding that AR was positively associated with intensity ratings and BL with pleasantness ratings for nuanced expressions supports the view that these two facets of interoception may play important roles in discriminative and affective processing, respectively. Discriminative processing should impact subjective ratings of interoceptive accuracy. For example, we would argue that by sustaining and controlling one’s attention to subtle signals (such as those arising from emotion contagion) one might amplify these cues, making them easier to monitor. In contrast, affective processing should play a key role in how we make interpersonal threat assessments, particularly when we are unsure how to proceed. In the present study, *low* BL scores predicted making more positive assessments of nuanced expressions, which predicted a stronger willingness to approach, and *high* BL scores were linked to making more negative assessments of these expressions, which predicted stronger hesitancy to approach. Given the roles that discriminative and affective processing are thought to play in important domains such as those mentioned above, learning more about the neural systems that support these types of processing should be an important focus of future research. The addition of objective measures of interoception may be particularly useful in this regard. Davenport and Vovk (2009) suggest that, in the respiratory domain, discriminative processing primarily involves somatosensory pathways, whereas affective processing/appraisal involves regions such as the amygdala, anterior cingulate, and insular cortex. This lends credence to Desmedt et al.’s (2023a) suggestion that it may be useful to expand the concept of interoception to include both somatosensory (exteroceptive/nonhomeostatic) processes (e.g., proprioception, nociception) and homeostatic processes.

In Study 2, we presented preliminary evidence that DIF was linked to difficulties with SR, a lack of trust in one’s body, the tendency to try to ignore or distract oneself from uncomfortable sensations (low ND), and (indirectly) with a heightened tendency to worry about or become emotionally distressed by uncomfortable sensations (low NW). Although we did not replicate the link between DIF and low ND in the present study (possibly due to low power), we did observe a trend for individuals scoring low in ND to rate nuanced expressions more positively than those who did not apply this strategy. This could reflect a form of avoidance coping, which has been linked to DIF in past research (e.g., Wood and Doughty, 2013). More work is needed to determine if/how problems with emotional appraisal (DIF/DDF) might influence not only coping mechanisms but also the development of dysfunctional beliefs about the cognitive, physical, or social impacts of unpleasant bodily sensations (such as those characteristic of anxiety sensitivity). It seems plausible that these beliefs could exacerbate the expression of SPS– traits by exerting top-down effects on how attentive we are to unpleasant bodily sensations and how much we worry about them.

One notable strength of the present study was the fact that we focused on nuanced expressions. It is crucial to expand our understanding of how viewers process and respond to such expressions, as most of the research in this area has focused on photographs of people displaying “basic” emotions (such as happiness, sadness, fear, and anger), captured at their apex. Because with strongly valenced emotional stimuli there is a near perfect correlation between valence and approach motivation, one must turn to the use of more nuanced stimuli to disentangle the influence of these constructs (Kaczmarek and Harmon-Jones, 2024). Additional research utilizing dynamic displays or live interactions and physiological recordings to explore individual differences in sensitivity to subtle changes in facial expressions, and to shifts between one expression (affective state) and another, is needed. Results from such studies may prove vital for theory building and could have important implications for mental health professionals working with individuals who experience difficulties with social perception and communication (e.g., Campbell et al., 2010; Frigerio et al., 2006; Schmolck et al., 2007).

Despite its strengths, we acknowledge that this study also had some limitations that may limit the conclusions that can be drawn. First, it might have been preferable to perform a preliminary norming study to identify nuanced expressions based on ratings obtained from a larger and independent sample. We will note, however, that the approach we took does increase confidence that members of our local sample viewed the target expressions as nuanced. As such, we feel our findings provide useful, preliminary insights into individual differences in personality and interoception that may have an impact on perceptual and social judgments.

A second limitation is that, given our sample size, we did not have sufficient power to test more complex relationships between our study variables. This was unavoidable, however, as testing had to be suspended during data collection due to university facility closures related to the COVID-19 pandemic.

Regarding our measure of approach motivation, in the interest of exploring prosocial tendencies it might have been informative to ask participants to rate how likely they would be to approach each person to offer them assistance, rather than to get their help. Nonetheless, past research suggests that the likelihood of making both types of judgments varies as a function of the other person’s facial expression and individual differences in emotional empathy (e.g., Willis et al., 2015). Finally, it might have been better to use an implicit measure of approach/avoidance tendencies (Kaczmarek and Harmon-Jones, 2024). For example, if we had used Rinck and Becker’s (2007) approach-avoidance task we could have compared the time it took participants to make behavioral push vs. pull responses when shown faces displaying nuanced expressions.

## 5 Conclusion

This set of studies makes several important contributions to the literature. First, our findings suggest a slightly different way of viewing EOT. We conceptualize it as a marker not only of the extent to which one fails to attend to one’s subjective *feelings* (Preece et al., 2017), but also to the specific exteroceptive and interoceptive cues that shape these experiences. We recognize that this is just one aspect of *la pensée opératoire* (Sifneos, 1973)—the cognitive style associated with EOT (see Taylor et al., 2023)—and that the degree to which one shows a preference for analytical reasoning may, at least partially, underlie some of the relationships we observed. Nonetheless, we wish to emphasize that, because levels of EOT can vary widely among those who meet traditional criteria for alexithymia (Jakobson et al., 2024; Jakobson and Rigby, 2021), it is important for alexithymia researchers to tease apart variance in interoception that may be related to EOT, rather than relying on global measures of alexithymia. Indeed, the global score on popular measures such as the TAS-20 or the Perth Alexithymia Questionnaire (Preece et al., 2018) is weighted toward problems with emotional appraisal, as these scales contain a disproportionate number of items assessing DIF and DDF. These problems seem to be related to how we cope with unpleasant sensations. However, the findings reported here suggest that levels of EOT may directly or indirectly influence subjective estimates of how accurately one can monitor their internal state (Study 1) and the extent to which one notices and regulates their attention to internal cues or processes how these cues relate to emotional states (Study 2), and listens to their body when making perceptual judgments about others’ expressions (Study 3). They also suggest that indirect links between EOT and these outcomes are frequently mediated by positive features of SPS. Importantly, this suggests that individuals who score *low* on EOT and *high* on SPS+ traits may not report difficulties in these domains of interoception, even if they score high on DIF and DDF (see also Jakobson and Rigby, 2021). Although causal attributions are unwarranted given the design of our studies and our relatively small samples, the preliminary findings reported here may guide the longitudinal or experimental studies that are needed to test directional hypotheses.

Our results also suggest a somewhat different framework for thinking about how different dimensions of subjective interoception may relate to one another. For example, rather than emphasizing links between the ND subscale and other measures of interoceptive attention, and between the NW subscale and other measures related to interoceptive interpretation (Desmedt et al., 2023a), it might be more important to emphasize how becoming overly focused on and worrying about uncomfortable or unpleasant sensations may contribute to top-down biases in interoceptive sensing (e.g., inflated estimates of how frequently painful sensations occur) and to the development of maladaptive beliefs (e.g., believing that the pain one is experiencing is permanent and debilitating). In short, exploring these ideas in future work may lead to advances in our understanding of how different aspects of interoception may contribute to a range of perceptual, cognitive, and emotional processes that can increase risk for a wide range of physical and mental health conditions. This work, in turn, may have important implications for tailoring individualized treatments for specific psychiatric disorders.

The current research was carried out in a non-clinical sample of young adults attending university and, as such, it will be important in future work to test the generalizability of these findings—particularly in clinical samples. Based on our preliminary results, however, we would urge clinicians to carefully consider individual differences in facets of alexithymia and SPS when tailoring their therapeutic approaches for individual clients. In particular, the results of Studies 1 and 2 support the importance of distinguishing between SPS+ and SPS– trait clusters. Past work suggests that, whereas the negative features appear to be particularly important predictors of poor mental health, the positive features are linked to enhanced social-affiliative sensitivity and other positive outcomes such as the tendency to focus on things that make one feel comfortable or provide pleasure (De Gucht et al., 2022), and resiliency (De Gucht et al., 2024; McQuarrie and Jakobson, 2025). SPS+ traits also include the tendencies to be creative, imaginative, and open to new experiences (Attary and Ghazizadeh, 2021). Future research might explore how multisensory integration, alexithymia, SPS, and interoception interact and impact these and other processes as well as prosocial action tendencies.

## Data Availability

The datasets presented in this study can be found in online repositories. The names of the repository/repositories and accession number(s) can be found at: “Interoception and Alexithymia” repository, https://doi.org/10.5683/SP3/YXGFKZ.

## Footnotes

1 The protocol for this study was identical to that employed by Jakobson and Rigby (2021), but there was no overlap between the participants in that study and the current sample.
